# QTL detection for bread wheat processing quality in a nested association mapping population of semi-wild and domesticated wheat varieties

**DOI:** 10.1186/s12870-022-03523-x

**Published:** 2022-03-21

**Authors:** Junmei Hu, Guilian Xiao, Peng Jiang, Yan Zhao, Guangxu Zhang, Xin Ma, Jie Yao, Lixia Xue, Peisen Su, Yinguang Bao

**Affiliations:** 1 State Key Laboratory of Crop Biology, Shandong Key Laboratory of Crop Biology, College of Agronomy, Shandong Agricultural University, Taian, 271018 The People’s Republic of China; 2 Lianyungang Academy of Agricultural Sciences, Lianyungang, 222000 The People’s Republic of China; 3 Yantai Academy of Agricultural Sciences in Shandong Province, Yantai, 265500 The People’s Republic of China; 4 Agricultural Technology Station, Sunwu Sub-district Office, Huimin County, Shandong Province 251700 Binzhou, The People’s Republic of China; 5 College of Agriculture, Liaocheng University, Liaocheng, 252059 The People’s Republic of China

**Keywords:** Wheat, Processing quality, Quantitative trait locus, Semi-wild wheat, Nested association mapping (NAM) population

## Abstract

**Background:**

Wheat processing quality is an important factor in evaluating overall wheat quality, and dough characteristics are important when assessing the processing quality of wheat. As a notable germplasm resource, semi-wild wheat has a key role in the study of wheat processing quality.

**Results:**

In this study, four dough rheological characteristics were collected in four environments using a nested association mapping (NAM) population consisting of semi-wild and domesticated wheat varieties to identify quantitative trait loci (QTL) for wheat processing quality. A total of 49 QTL for wheat processing quality were detected, explaining 0.36–10.82% of the phenotypic variation. These QTL were located on all wheat chromosomes except for 2D, 3A, 3D, 6B, 6D and 7D. Compared to previous studies, 29 QTL were newly identified. Four novel QTL, *QMlPH-1B.4*, *QMlPH-3B.4*, *QWdEm-1B.2* and *QWdEm-3B.2*, were stably identified in three or more environments, among which *QMlPH-3B.4* was a major QTL. Moreover, eight important genetic regions for wheat processing quality were identified on chromosomes 1B, 3B and 4D, which showed pleiotropy for dough characteristics. In addition, out of 49 QTL, 15 favorable alleles came from three semi-wild parents, suggesting that the QTL alleles provided by the semi-wild parent were not utilized in domesticated varieties.

**Conclusions:**

The results show that semi-wild wheat varieties can enrich the existing wheat gene pool and provide broader variation resources for wheat genetic research.

**Supplementary Information:**

The online version contains supplementary material available at 10.1186/s12870-022-03523-x.

## Background

Bread wheat (*Triticum aestivum* L*.*) is a crucial source of protein, minerals and vitamins that feeds over 35% of the world’s population [1, 2]. Hence, improving the processing quality of wheat is an important goal in wheat breeding. Investigation of the relationship between seed storage protein alleles and processing characteristics indicates that storage proteins are the main determinant of wheat processing quality [3]. The seed storage proteins in wheat consist of gliadin and glutenin, which can be used to predict dough rheological properties, including the viscoelastic and mixing properties [4]. The genes coding for gliadin are located on the short arms of the chromosomes of homologous groups 1 and 6 [5]. Glutenin in the endosperm consists of high-molecular-weight (HMW-GS) and low-molecular-weight glutenin subunits (LMW-GS). HMW-GSs are encoded by *Glu-1* loci that are located on the long arms of the chromosomes of homologous group 1, including *Glu-A1*, *Glu-B1* and *Glu-D1* [5]. LMW-GSs are encoded by *Glu-3* loci that are located on the short arms of the chromosomes of group 1, including *Glu-A3*, *Glu-B3* and *Glu-D3* [6]. Research has indicated that both glutenin and gliadin are significantly associated with wheat processing quality by affecting the viscoelasticity and flexibility of dough [7, 8].

Most of the quality-related traits of interest in wheat breeding are characterized by polygenic inheritance, which is generally studied with quantitative trait loci (QTL) mapping [3, 9–16]. Some stable QTL for protein content were discovered on chromosomes 1A, 1D, 2B, 3A, 4A, 5A, 5D, 7A, and 7B [3, 7, 12, 15, 17, 18]. Krystkowiak et al. [7] detected one major QTL on chromosome 5D that influences starch content, wet gluten content, and zeleny sedimentation value. Stable QTL for the starch content of wheat flour were detected on chromosomes 4A and 7D [9]. In addition, QTL for wet gluten content of wheat flour were identified on chromosomes 5AS and 5AL [15].

Due to a complex interaction between proteins and other components, such as pentosans, the predictability of dough strength from chemical composition is difficult, and therefore rheological tests are required [19, 20]. The dough rheological characteristics of wheat are quantitative traits that are dependent on multiple genes and are greatly influenced by environmental conditions. Studies have reported that dough properties are influenced by the properties of storage proteins, which can be reflected by mixograph, farinograph, and extensograph parameters [13, 19, 21]. Many rheological tests have been widely used as predictors of wheat processing quality and end-use quality [3, 15, 19]. Mann et al. [3] discovered that dough rheology QTL were highly correlated across multiple environments and primarily influenced by the *Glu-1* loci (*Glu-B1*, *Glu-D1*). Tsilo et al. [22] detected a major QTL cluster for dough rheological properties on chromosome 1B, which explained the large total phenotypic variation in dough development time, mixing tolerance index, dough stability and time to dough breakdown.

Several segregant biparental populations can be adopted for QTL mapping, such as backcross, F_2_, doubled haploid (DH), introgression lines and recombinant inbred line populations. In most previous studies, these approaches for wheat processing quality QTL mapping were utilized [9, 10, 23]. However, the linkage analysis achievable with bi-parental populations showed a narrow genetic background and was often able to detect QTL only with large intervals because of limited recombination events [24–26]. This limitation can be partially overcome by analyzing multiple related populations, such as nested association mapping (NAM) population [27, 28]. The NAM population is a composite population composed of multi-family recombinant inbred line population constructed by the hybridization of one common parent with several other parents and multi-generation continuous self-cross. Due to its wide genetic diversity and high resolution, NAM population is an ideal population for QTL analysis. Such populations have been used to identify QTL in different crops, including maize, soybean, sorghum, barley, bread wheat and durum wheat [29, 30]. However, there are few studies on QTL analysis of wheat processing quality using a NAM population [31, 32].

In previous studies, most of the material on wheat processing quality represented domesticated cultivars, and compared with wild and semi-wild varieties, their genetic diversity will decrease with domestication [33]. Through whole-genome sequencing analysis, it was determined that composite introgression from wild populations contributed to 4–32% of the bread wheat genome, which increased the genetic diversity of bread wheat [34]. Three semi-wild wheat subspecies germplasm resources unique to western China, including the Tibetan weedrace (*T. aestivum* ssp. *tibetanum* Shao) characterized by strong seed dormancy, hulled glumes and brittle spikelets, Xinjiang rice wheat (*T. petropavlovskyi* Udats. et Migusch.) characterized by a long glume, and Yunnan hulled wheat, or “Tiekemai” (*T. aestivum* ssp. *yunnanense* King), named for its very hard and tough glumes that adhere to the grains [35–37]. The semi-wild wheat subspecies in China have a primitive chromosomal constitution, which is important to probe the effect of domestication on processing quality in wheat breeding [35]. Therefore, in this study, a wheat NAM population was constructed by crossing one common parent, Yanzhan 1, and four divergent parents, including three semi-wild cultivars from China and one domesticated variety from the British islands. This NAM population was used for QTL mapping for wheat processing quality, which will facilitate high-quality wheat breeding and marker-assisted selection (MAS) in wheat breeding.

## Results

### Phenotypic analysis of wheat processing quality

**Fig. 1:**
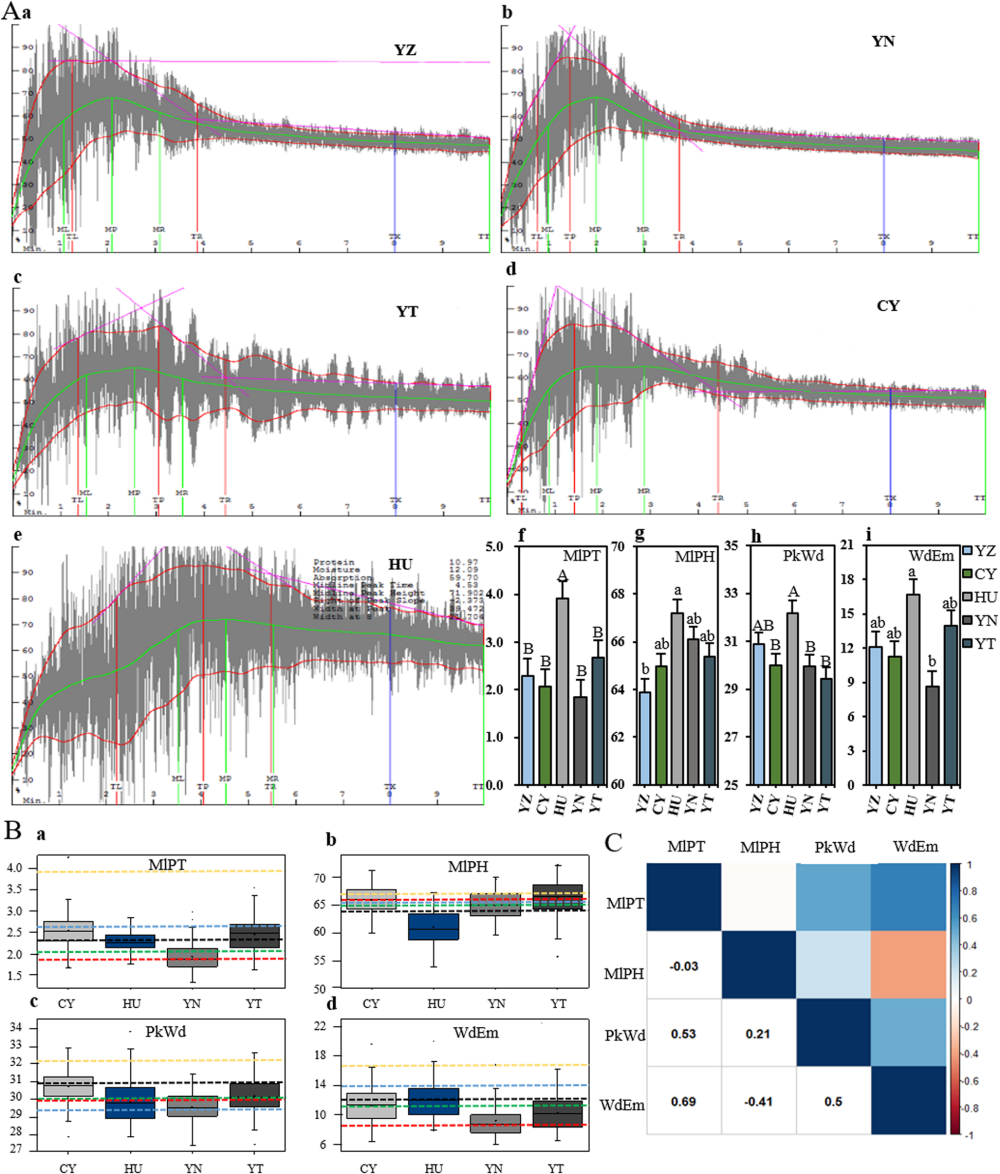
Phenotypic data of wheat processing quality of the five parents and the NAM population. **A** The four dough rheological characteristics of the five parents (a–e) and the ANOVA between five parents of four dough rheological characteristics (f–i). Labels A and B indicate significant differences at the level of *P* < 0.01, and labels a and b indicate significant differences at the level of *P* < 0.05. **B** The boxplot for four dough rheological characteristics of four RIL populations. The different color lines of black, green, yellow, red, and blue indicate the five parents of the NAM population YZ, CY, HU, YN, and YT, respectively. **C** The relationships between four dough rheological characteristics of the NAM population. YZ, Yanzhan 1; CY, Chayazheda 29; HU, Hussar; YN, Yunnanxiaomai; YT, Yutiandaomai. MlPT, midline peak time; MlPH, midline peak height; PkWd, peak width; WdEm, width at eight minutes

The five parents of the NAM population had different dough rheological characteristics (Fig. 1A). Among them, HU (Hussar) had the best dough characteristics, while the dough characteristics of YZ (Yanzhan 1) and YN (Yunnanxiaomai) were poor. We found that the common parent YZ had a longer MlPT (midline peak time), wider PkWd (peak width) and wider WdEm (width at eight minutes) than the other parents, CY (Chayazheda 29) and YN, and had the smallest MlPH (midline peak height) compared with the other four donor parents. HU had the highest values for all dough rheological characteristics compared with the other four parents, which indicated that HU had the best dough characteristics of the individual’s studies here. CY had a longer MlPT, wider PkWd and wider WdEm than YN. YN had a wider MlPH than YZ, CY and YT but the shortest MlPT and the narrowest WdEm compared with the other four parents. Compared with YZ, CY and YN, YT (Yutiandaomai) had the longest MlPT, widest WdEm and narrowest PkWd. Observing the phenotypic data distribution of the NAM population, there was strong transgressive segregation for all dough rheological characteristics in each RIL population except for the MlPT of HU-RILs (Fig. 1B, Table S1).

To evaluate the pairwise correlations between dough rheological characteristics, Pearson’s correlation was estimated using BLUP (best linear unbiased prediction) values combined over four environments (Fig. 1C). WdEm was significantly positively correlated with MlPT and PkWd but significantly negatively correlated with MlPH. PkWd was significantly positively correlated with MlPT and MlPH. The correlation between MlPT and MlPH was not significant.

The heritabilities of dough rheological characteristics in the NAM population were 42.7–84.7%, and they were differed largely in the four RIL populations (Table S2). PKWD is a relatively important dough rheological parameter to measure wheat processing quality, and its phenotype is greatly affected by the environment (42.7 –59.7%). Among them, the heritabilities of the HU-RIL and YT-RIL populations were higher, whereas those in the YN-RIL population were lower.

### QTL analysis of wheat processing quality

**Fig. 2:**
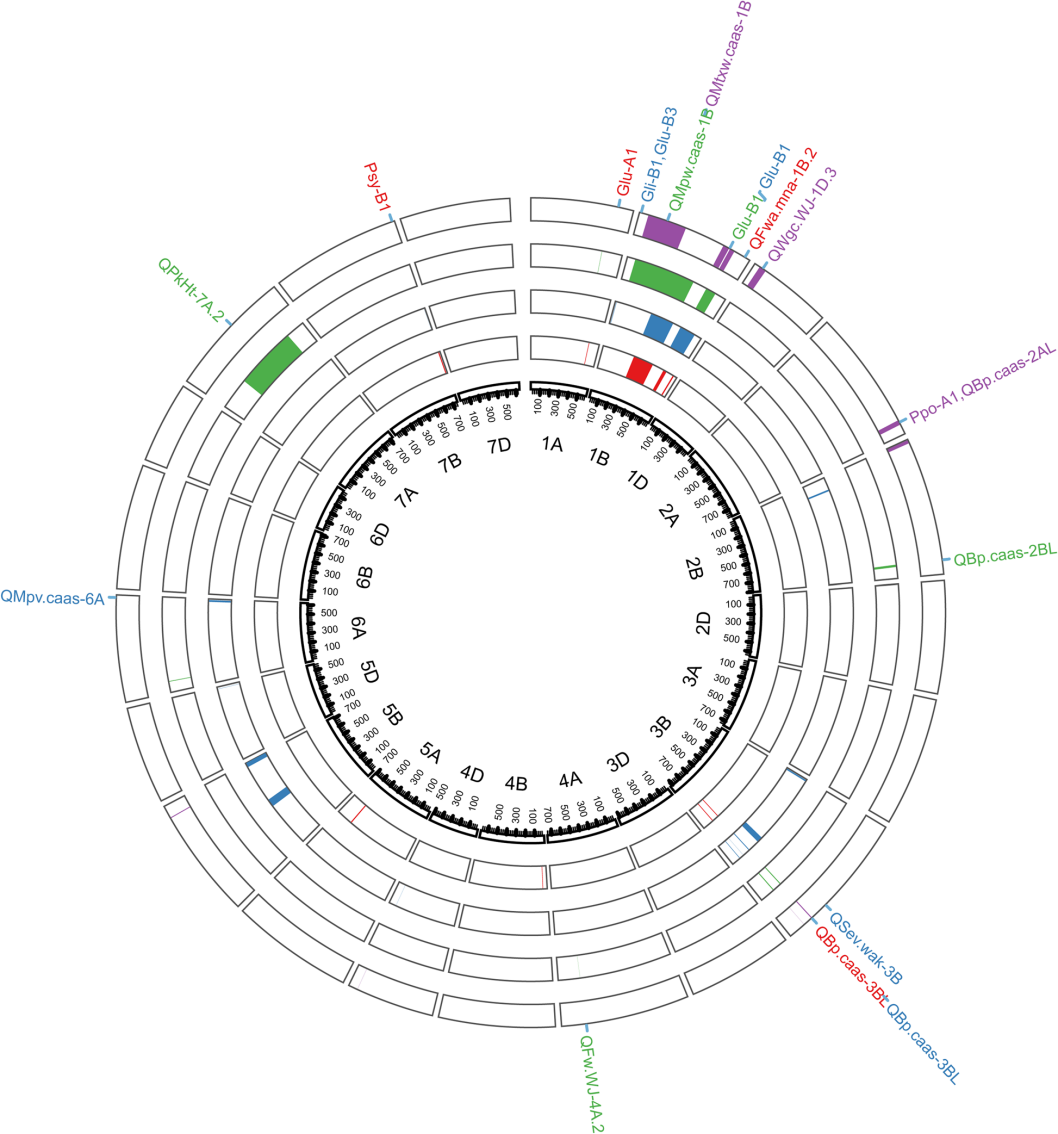
Identified QTL for wheat processing quality detected in the NAM population. Red, blue, green and purple indicate the midline peak time, the midline peak height, the peak width, and the width at eight minutes, respectively. The outermost part of the graph represents the QTL/gene reported in the previous study

A total of 49 QTL were detected on chromosomes 1A (2), 1B (17), 1D, 2A, 2B (3), 3B (11), 4A, 4B, 4D (2), 5A, 5B (3), 5D, 6A (2), 7A, and 7B (2) for wheat processing quality in four individual environments and combined QTL analysis (Fig. 2, Table S3). Ten, eighteen, eleven, and ten QTL were identified for MlPT, MlPH, PkWd and WdEm, respectively. These QTL explained 0.36–10.82% of the phenotypic variation. Thirty-four of these QTL were identified in the individual environment and the combined environment analysis. The favorable alleles of two, seven, six, ten and twenty-four QTL were provided by parents CY, YN, YT, HU and YZ, respectively (Table S4).

For MlPT, ten QTL were found on chromosomes 1A, 1B (4), 3B (2), 4B, 5A, and 7B in four environments and the combined environment analysis, explaining a range of 1.47% to 8.29% of the phenotypic variation (Fig. 2, Table S3). Six of those QTL were detected in the individual environment and combined environment analyses. One stable QTL, *QMlPT-1B.2*, had a favorable allele from YT and was found in two individual environments and the combined environment analysis, explaining 2.20–3.66% of the phenotypic variation. The donor parent YN contributed the best favorable allele for *QMlPT-3B.1*, which was stably detected in three individual environments, explaining 2.86–4.14% of the phenotypic variation. Five QTL, including *QMlPT-1A*, *QMlPT-1B.3*, *QMlPT-1B.4*, *QMlPT-4B* and *QMlPT-7B*, were identified in one environment and the combined environment analysis, with 4.15–8.29%, 2.80–3.28%, 3.31–4.19%, 2.74–3.11% and 1.47–4.02% of the phenotypic variation, respectively. *QMlPT-1B.1*, *QMlPT-3B.2* and *QMlPT-5A*, with LOD values of 3.71–6.38, 3.08–8.03 and 2.66–4.88, respectively, were found in two environments, accounting for 2.24–5.42%, 2.07–3.70% and 1.78–1.82% of the phenotypic variation, respectively. The favorable alleles of two and three QTL of the ten QTL were provided by semi-wild parents YN and YT, respectively, while one and four QTL out of the remaining QTL were provided by domesticated parents HU and YZ, respectively (Table S4).

For MlPH, 18 QTL were identified, which were distributed on chromosomes 1B (6), 2B, 3B (5), 4D, 5B (2), 5D, 6A, and 7B in four environments and the combined environment analysis, explaining 0.36–10.82% of the phenotypic variation (Fig. 2, Table S3). Twelve of the eighteen QTL were detected in both individual environments and the combined environment analysis. The favorable alleles of two stable QTL (*QMlPH-3B.3* and *QMlPH-3B.4*) were contributed by HU, which were identified in all four environments and the combined environment analysis, explaining 1.50–8.60% and 1.71–10.82% of the phenotypic variation, respectively. *QMlPH-1B.4*, a favorable allele from the common parent YZ, was stably identified in four environments, with an LOD value of 4.01–9.75 and phenotypic variation of 1.42–4.50%. *QMlPH-1B.3* and *QMlPH-5D*, with LOD values of 5.18–10.25 and 3.21–5.99, respectively, were stably detected in the two environments and the combined environment analysis, accounting for 1.15–4.90% and 1.10–2.21% of the phenotypic variation, respectively. Eight QTL, including *QMlPH-1B.5*, *QMlPH-1B.6*, *QMlPH-3B.1*, *QMlPH-4D*, *QMlPH-5B.1*, *QMlPH-5B.2*, *QMlPH-6A* and *QMlPH-7B*, were detected in one environment and the combined environment analysis. In addition, five QTL, *QMlPH-1B.1*, *QMlPH-1B.2*, *QMlPH-2B*, *QMlPH-3B.2* and *QMlPH-3B.5*, were identified in two environments. Three OTL with favorable alleles were detected in the three semi-wild parents CY, YN, and YT (Table S4). Compared with the other four parents, the alleles of the domesticated parent HU increased the MlPH for five QTL, while the common parent YZ decreased the MlPH for ten QTL.

For PkWd, eleven QTL were stably detected on chromosomes 1A, 1B (4), 2B, 3B (2), 4A, 6A, and 7A, explaining 0.55–6.09% of the phenotypic variation (Fig. 2, Table S3). All of these QTL except for *QPkWd-1B.1* were identified in both individual environments and the combined environment analysis. *QPkWd-3B.1* and *QPkWd-3B.2* are favorable alleles from the common parent YZ and were detected in three or more environments. For five QTL (*QPkWd-1B.2*, *QPkWd-1B.3*, *QPkWd-1B.4*, *QPkWd-4A* and *QPkWd-6A*), the alleles increasing PkWd were provided by YZ, accounting for 1.74–5.31%, 1.85–4.70%, 1.93–4.85%, 0.76–3.02% and 1.49–3.37% of the phenotypic variation, respectively. For *QPkWd-1A* and *QPkWd-7A*, the alleles increasing PkWd were donated by HU, with 5.85–7.29 and 3.04–3.36 of the LOD value, respectively, accounting for 2.15–2.81% and 1.12–2.88% of the phenotypic variation, respectively. In addition, in *QPkWd-1B.1* and *QPkWd-2B*, the alleles decreasing PkWd were provided by CY and YT, respectively, accounting for 0.96–4.80% and 0.55–4.16% of the phenotypic variation, respectively.

For WdEm, ten QTL were detected on chromosomes 1B (3), 1D, 2A, 2B, 3B (2), 4D, and 5B in individual environmental and combined environment analyses, accounting for 0.51–9.70% of the phenotypic variation (Fig. 2, Table S3). Six QTL were identified in both individual environments and the combined environment analysis. Two QTL, *QWdEm-1B.2* and *QWdEm-3B.1*, were stably identified in three environments and the combined environment analysis, with 4.70–5.78 and 4.44–16.13 of the LOD value, respectively, explaining 0.51–2.42% and 2.14–8.82% of the phenotypic variation, respectively. *QWdEm-1B.1* and *QWdEm-1B.3* had LOD values of 4.14–4.89 and 4.87–6.87, respectively and were detected in the two environments and the combined environment analysis. In these two QTL, the alleles decreasing WdEm were donated by YN, accounting for 1.19–2.73% and 1.75–3.25% of the phenotypic variation, respectively. The alleles in HU increased WdEm for two QTL, *QWdEm-2A* and *QWdEm-4D*, which explained 2.34–4.05% and 0.85–2.12% of the phenotypic variation, respectively. For *QWdEm-1D* and *QWdEm-2B*, favorable alleles were donated by YT and YN, respectively. Favorable alleles of three QTL, *QWdEm-3B.1*, *QWdEm-3B.2*, and *QWdEm-5B*, were provided by the common parent YZ. Among the ten QTL for WdEm, the favorable alleles of four and one QTL were provided by semi-wild parents YN and YT, respectively. In addition, two and three QTL were provided by domesticated parents HU and YZ, respectively (Table S4).

### Eight important genetic regions for wheat processing quality

In this study, eight important genetic regions were found on chromosomes 1B (4), 3B (3), and 4D (Table 1). *QG-3B.1* was associated with all dough rheological characteristics, *QMlPT-3B.1*, *QMlPH-3B.3*, *QPkWd-3B.1*, and *QWdEm-3B.1*, within 4.71 Mb of physical distance. Three genetic regions, *QG-1B.3*, *QG-3B.2*, and *QG-4D*, influenced MlPH and WdEm. *QG-1B.2*, located on flank marker *BS00047700_51*–*IAAV4866*, was associated with MlPT, MlPH, and PkWd. *QG-1B.4*, located on flank marker *tplb0048b10_1365*–*Ku_c28580_432*, influences dough rheological characteristics MlPT, MlPH, and WdEm. In addition, *QG-1B.1* and *QG-3B.3* were located on flank markers *wsnp_Ku_rep_c70742_70379526*–*tplb0059c20_2221* and *wsnp_Ex_c64005_62987067*–*wsnp_BE497740B_Ta_2_1*, respectively, which influence PkWd and WdEm, and PkWd and MlPT, respectively.

**Table 1:** Eight important genetic regions associated with multiple dough rheological characteristics

Genetic region	QTL	Flanking markers of peak	
*QG-1B.1*	*QPkWd-1B.1*, *QWdEm-1B.1*	*wsnp_Ku_rep_c70742_70379526*	*tplb0059c20_2221*
*QG-1B.2*	*QMlPT-1B.1*, *QMlPH-1B.2*, *QPkWd-1B.2*	*BS00047700_51*	*IAAV4866*
*QG-1B.3*	*QWdEm-1B.2*, *QMlPH-1B.3*	*Tdurum_contig20299_368*	*BobWhite_c48550_198*
*QG-1B.4*	*QMlPT-1B.2*, *QWdEm-1B.3, QMlPH-1B.4*	*tplb0048b10_1365*	*Ku_c28580_432*
*QG-3B.1*	*QMlPT-3B.1*, *QPkWd-3B.1*, *QWdEm-3B.1*, *QMlPH-3B.3*	*wsnp_Ex_c3257_6003626*	*wsnp_Ex_c8715_14590273*
*QG-3B.2*	*QWdEm-3B.2*, *QMlPH-3B.4*	*wsnp_Ku_c29102_39008953*	*wsnp_Ex_c64005_62986957*
*QG-3B.3*	*QMlPT-3B.2*, *QPkWd-3B.2*	*wsnp_Ex_c64005_62987067*	*wsnp_BE497740B_Ta_2_1*
*QG-4D*	*QMlPH-4D*, *QWdEm-4D*	*BS00103682_51*	*Ex_c42133_630*

*MlPT* Midline Peak Time, *MlPH* Midline Peak Height, *PkWd* Peak Width, *WdEm* Width at Eight minutes, *QG* QTL cluster

## Discussion

### Trait correlations

Wheat processing quality is a quantitative genetic trait controlled by multiple genes and affected by the environment. The dough characteristics influenced by glutelin and gliadin are comprehensive traits that reflect wheat processing quality. In this study, a NAM population consisting of three unique semi-wild wheat cultivars of China (CY, YN, and YT), the Chinese domesticated cultivar YZ, and the British domesticated cultivar HU was used to identify QTL regulating wheat processing quality. The small size of a single RIL population that constitutes the NAM population leads to fewer recombination events among parents and limits the precise locations of QTL. Even the NAM population based on polymorphism between YZ and the other four parents contains a series of recombinants with broader genetic bases, more populations are required to improve the mapping resolution and increase the number of QTL detected. In this study, the genetic map was generated by using 90 K SNP array. With the rapid development of sequencing and gene-chip technologies, new generation of high-density SNP chip [38] or genome re-sequencing [39] can offer high-resolution genetic map, by which more QTL related to wheat processing quality are supposed to be identified.

Here, we found that the dough rheological characteristics were different among three semi-wild wheat varieties (YN, YT, CY) and two cultivated wheat cultivars (HU and YZ) (Fig. 1A). Therefore, the NAM population composed of the five parents was used to detect QTL of wheat processing quality. The results show that the NAM population had large variation between three semi-wild RIL populations, CY-RILs, YN-RILs, and YT-RILs (Fig. 1B).

The difference between MlPT and MlPH was not significant, but there were significant correlations between the other dough rheological characteristics (Fig. 1C). Moreover, one important genetic region, *QG-3B.1*, associated with all dough rheological characteristics, was detected (Table 1). In addition, in terms of the association among the four dough rheological characteristics, MlPT was significantly positively correlated with PkWd and WdEm (Fig. 1C). Specifically, PkWd and WdEm increased with increasing MlPT (verified by *QG-1B.2, QG-1B.4* and *QG-3B.3*; Fig. 1B-C, Table 1). Although MlPT and MlPH were colocalized between two genetic regions, *QG-1B.2* and *QG-1B.4*, the correlation between MlPT and MlPH was not significant. We suspect that this may be because MlPT is related to the protein content, while MlPH is related to the gluten strength and the ability of the dough to resist external forces. WdEm was significantly positively correlated with PkWd (verified by *QG-1B.1*) and significantly negatively correlated with MlPH (substantiated by *QG-1B.3*, *QG-3B.2*, and *QG-4D*, Fig. 1C, Table 1).

### Comparison with previous studies

In this study, 49 QTL for wheat processing quality were identified, 29 of which were unique to this study compared with previous studies, and four novel QTL (*QMlPH-1B.4*, *QMlPH-3B.4*, *QWdEm-1B.2* and *QWdEm-3B.2*) were stably identified in three or more environments (Fig. 2, Tables 2, S3). For MlPT, five of ten QTL were previously reported. *QMlPT-1A*, with a favorable allele in HU, was mapped close to the gene *Glu-A1*, whose effect was consistent with the longer MlPT of Hussar (Figs. 1A, 2, Table S3) [40]. Similar genetic regions of two QTL, *QMlPT-1B.3* and *QMlPT-1B.4*, which had favorable alleles from the common parent YZ, were reported by Tsilo et al. [22] (Fig. 2, Table S3). *QMlPT-3B.1* was previously reported by Liu et al. [11] to be located at a similar genetic region on chromosome 3B. *QMlPT-7B* was identified close to the *Psy-B1* gene, which indicates that *Psy-B1* is not only associated with the synthesis of carotenoids, but might affect the wheat processing quality [41].

**Table 2:** Four novel QTL that were stably identified in three or more environments

QTL	Environment	Position	Flank markers of peak		LOD	PVE (%)
*QMlPH-1B.4*	E1/E2/E3/E4	44	*tplb0048b10_1365*	*Ku_c28580_432*	4.0129–9.7453	1.4168–4.503
*QMlPH-3B.4*	E1/E2/E3/E4/BLUP	63	*wsnp_Ku_c29102_39008953*	*wsnp_Ex_c64005_62986957*	6.365–32.0377	1.711–10.8244
*QWdEm-1B.2*	E1/E2/E3/BLUP	38	*Tdurum_contig20299_368*	*BobWhite_c48550_198*	4.6957–5.7802	0.5107–2.4201
*QWdEm-3B.2*	E1/E2/E3	63	*wsnp_Ku_c29102_39008953*	*wsnp_Ex_c64005_62986957*	6.1816–20.4799	2.5598–9.7032

*MlPT* Midline Peak Time, *MlPH* Midline Peak Height, *PkWd* Peak Width, *WdEm* Width at Eight minutes. *E1* 2016 Dezhou, *E2* 2016 Tai’an, *E3* 2016 Heze, *E4* 2017 Tai’an, *BLUP* Best Linear Unbiased Prediction

Eighteen QTL for MlPH were identified, five of which were previously reported (Fig. 2, Table S3). *QMlPH-1B.1* was detected in two environments and was located near two genes, *Gli-B1* and *Glu-B3*. *QMlPH-1B.5* was located near gene *Glu-B1* [42]. Three QTL, *QMlPH-3B.2*, *QMlPH-3B.3*, and *QMlPH-6A*, were detected at a similar genetic region by Carter et al. [43], Liu et al. [11], and Li et al. [23], respectively. Among the five QTL that were reported in previous studies, the allele of *QMlPH-3B.3* was provided by HU with higher MlPH, and favorable alleles of the other QTL were detected in YZ with lower MlPH, indicating that YZ may have a recessive allelic variation gene that affects gluten strength (Fig. 1A, Table S3).

Regarding PkWd, eleven QTL were detected, six of which were reported by previous studies (Fig. 2, Table S3). Two QTL, *QPkWd-1B.1* and *QPkWd-4A*, at similar genetic regions were reported by Li et al. [23, 44]. *QPkWd-1B.3* was mapped close to the gene *Glu-B1* [42]. Two QTL, *QPkWd-2B* and *QPkWd-3B.1*, were previously reported by Liu et al. [11] in a similar genetic region. In addition, *QPkWd-7A* was located at a similar genetic region in a previous study by Zhang et al. [15].

Regarding WdEm, four of ten QTL were detected in previous studies (Fig. 2, Table S3). Three QTL, *QWdEm-1B.1*, *QWdEm-1D*, and *QWdEm-3B.1*, were reported from similar genetic regions on chromosomes 1B, 1D, and 3B, respectively [11, 23, 44]. In addition, *QWdEm-2A* was previously reported at a similar genetic region [11, 45, 46].

### Application potential of semi-wild cultivars in breeding good-quality wheat

Four novel QTL, *QMlPH-1B.4*, *QMlPH-3B.4*, *QWdEm-1B.2*, and *QWdEm-3B.2*, were stably detected (Table 2). One novel and major QTL, *QMlPH-3B.4*, was detected in all four environments and the combined QTL analysis, and its favorable allele came from the good-quality parent HU. Domesticated parent HU had the longest MlPT, highest MlPH, and widest PkWd and WdEm among the parents (Fig. 1A). *QMlPH-3B.4* could be utilized for quality improvement of YZ by increasing MlPT, MlPH, PkWd, and WdEm. We generally think that QTL detected in multiple environments should also be detectable when using BLUP values. *QMlPH-1B.4* was detected in four environments except for BLUP. This may be because the BLUP value only considers the contribution of genetic factors to the phenotype. Therefore, we think that environmental effects may explain why that QTL was not detected under BLUP. In summary, to achieve good-quality wheat production, breeding wheat varieties that contain high-quality genotypes is paramount, but a suitable planting environment is also necessary [47].

Forty-nine QTL were identified in this study, of which 23 were identified in one environment and combined QTL analysis. Among the 23 QTL, five, eight, eight and two QTL were for MlPT, MlPH, PkWd, and WdEm, respectively (Table S3). For PkWd, 73% of QTL were detected in one environment and combined QTL analysis, which could be because PkWd is highly influenced by the environment. This phenomenon can be verified by the heritability of PkWd, which has the lowest heritability among the four dough rheological characteristics (Tables S2). Among the 49 QTL, we found that favorable alleles of 17 QTL located on chromosome 1B were provided by YZ, CY, YN, and YT, while favorable alleles of nine QTL located on chromosome 3B were detected in domesticated parents HU and YZ (Table S3). Four Chinese cultivars have poor dough characteristics compared with the British domesticated cultivar HU (Fig. 1A). This phenomenon suggests that there may be genes on chromosome 3B of HU that affect wheat processing quality.

The existing gene pool of cultivated wheat is relatively narrow because it is composed of current and historical wheat cultivars lacking allelic variation from landraces and wild species [23, 33]. HMW-GS plays an important role in influencing dough processing quality and extensive studies have attempted to explore novel alleles of HMW-GS from wheat wild species as well as their potential application in breeding [48–50]. Recently, Talini et al. showed that *Triticum urartu*, a wild diploid wheat, present a series of new types of HMW-GS with improved flour quality than the cultivated materials [48]. The wheat relatives of *Aegilops umbellulata* and *Aegilops searsii* were also shown to have novel HMW-GS alleles different from common wheat which can be important resources for improving wheat processing quality [49, 50]. However, more genetic variations affecting process quality other than HMW-GS loci is still encouraged to be explored from wheat wild species and its relatives [48, 51].

The latest research shows that from the perspective of the whole genome level, Tibetan semi-wild wheat has been de-domesticated from local landraces, and its genome is rich in variation [52]. Therefore, as a valuable resource to broaden the genetic diversity of wheat breeding, Chinese semi-wild cultivars can be used for genetic research of wheat processing quality, especially the release of the semi-wild wheat reference genome (Tibetan semi-wild wheat) [52]. The favorable alleles of 31% of the QTL for wheat processing quality were provided by three semi-wild parents, which may be because semi-wild wheat contains alleles that are not utilized by existing cultivated wheat varieties [36, 53] (Table S4). Hence, semi-wild wheat varieties can enrich the existing wheat gene pool, provide broader resources for wheat genetic research, and help in investigating the effect of domestication on the processing quality of wheat.

## Conclusions

A wheat NAM population consisting of semi-wild and domesticated wheat varieties was used to detect QTL for wheat processing quality. A total of 49 QTL were identified, of which four novel QTL were stably identified in three or more environments. In addition, 15 of 49 QTL favorable alleles were provided by three semi-wild parents, which indicated that semi-wild wheat contained unique genetic material that was not used in domesticated varieties. Therefore, semi-wild wheat can be used as a genetic resource to enrich the existing wheat gene pool and provide more abundant variation for genetic research on wheat processing quality. In addition, the release of whole genome data of semi-wild wheat (Tibetan semi-wild wheat) [52] provides genomic information for further discovery of excellent alleles in semi-wild wheat and highlights the significance of studying the role of semi-wild wheat in evolution.

## Methods

### Plant material and experimental design

Previously, a wheat NAM population consisting of thirty-four RIL populations was constructed with Yanzhan 1 (YZ, *T. aestivum* L.) from Henan Province of the Huanghuai region, China as the common parent. All of the RIL populations (nine and ten generations of self-pollination) were derived using a single seed descent method. Here, to detect potential genetic alleles regulating wheat processing quality from broader genetic background, we selected four RIL populations for the NAM based QTL identification. Three of four divergent parents were semi-wild cultivars in China, including Yunnanxiaomai (YN, *T. aestivum* ssp*. yunnanense* King) from Yunnan Province [36], Yutiandaomai (YT, *T. petropavlovskyi* Udats. et Migusch.) from Sinkiang, and Cayazheda 29 (CY, *T. aestivum* ssp*. tibetanum* Shao) from Tibet [37, 52]. The other divergent parent was the British dwarf cultivar Hussar (HU), which is a good-quality cultivar [54]. The hybridizations of YZ with YN, YT, CY, and HU ultimately yielded 98, 93, 82, and 97 lines, respectively.

The NAM population along with the five parents were planted in De’zhou (E1, 116.39°E, 37.38°N), Tai’an (E2, 117.17°E, 36.17°N) and He’ze (E3, 115.50°E, 35.57°N) in Shandong Province during 2015–2016. The materials were planted again in Tai’an (E4) during 2016–2017. In each environment, each plot comprised two rows with a 2.0 m row length, 0.25 m row spacing, and 50 seeds per row. Two replicates were performed under each environment. All fields were managed in accordance with standard local practices.

### Traits investigated

The plants in each plot were harvested to evaluate the wheat processing quality. The moisture (%) and protein (%) of grain and flour of the NAM population were determined by near-infrared reflectance spectroscopy on a Perten Diode Array 7200 (Perten Instruments, Huddinge, Sweden) instrument according to the methods of the American Association of Cereal Chemists (AACC) 39–10 and 39–11 [55]. The grains were conditioned to 14% moisture content and then milled using a Quadrumat Junior (Brabender GmbH & Co. KG Duisburg, Germany.) according to the methods of AACC 26–95 and 26–50. Then, a 10 g-Mixograph (National Mfg. Co. Nebraska, America) was used to carry out rheological tests according to the AACC 54-40A method. For each of the samples, two planting replications were used for phenotypic data collection, and the following parameters were recorded: MlPT, midline peak time (min); MlPH, midline peak height (%); PkWd, peak width (%); and WdEm, width at eight minutes (%).

### Statistical analysis of phenotypic data

The best linear unbiased prediction (BLUP) for each line of wheat processing quality was counted across environments using the “lmer” function implemented in the R package lme4 (https://cran.r-project.org/web/packages/lme4/index.html). Each BLUP was used to calculate the pairwise correlations for phenotypic data using the “rcorr” function implemented in the R package Hmisc. Boxplots for phenotypic data were obtained from the BLUP value using Origin Pro V9.1 software (https://www.originlab.com/). Analysis of variance (ANOVA) for the five parents in wheat processing quality was calculated by Statistics Program for Social Sciences V20 software. Furthermore, the heritabilities were calculated through the AOV function of IciMapping V4.1 software using the formula *h*^2^ = *V*_*G*_ / (*V*_*G*_ + *V*_GEI_ /*e* + *V*_*e*_/*er*), where *V*_G_, *V*_GEI_ and *V*_e_ are the variances of *G* (genotypes), *GEI* (genotype × environment interactions) and the error, respectively; *e* is the number of environments; and *r* is the number of replications [56].

### QTL analysis

An integrated high-density linkage map (containing 2009 SNP markers) published previously was used in this study [57], and the procedure was as follows: first, the redundant markers of the 90,000 SNP array were processed through the “BIN” function of IciMapping V4.1. Second, the remaining markers were divided into different linkage groups through the “MAP” function of IciMapping V4.1 [56]. Third, based on the Kosambi mapping function, we constructed four individual maps of the RILs. Finally, four individual maps were combined with Join Map V4.0 (https://www.kyazma.nl/index.php/JoinMap/). The averaged value for each line in each environment was used to conduct individual environment QTL analysis, and BLUP values across four environments for each line were used for combined QTL analysis. QTL detection for wheat processing quality was performed by joint inclusive composite interval mapping (JICIM) in IciMapping V4.1 software [56]. Using this method, the walking step was set as 1.0 cM, and a stepwise regression probability of 0.001 was used to identify QTL. A QTL was identified when the LOD score was greater than 2.5 in the NAM population and greater than 2.0 in at least one RIL population. In this study, QTL clusters affecting quality-related traits (MlPT, MlPH, PkWd, and WdEm) were defined with the prefix “*QG*”.

## Supplementary Information

**Additional file 1:** **Table S1.** Phenotype data of five parents in four environments and of the NAM population in individual environments and combined environments.

**Additional file 2:** **Table S2. **ANOVA analysis of quality-related traits for the NAM population.

**Additional file 3:** **Table S3**. The QTL mapping results for four dough rheological characteristics in the individual environment analysis and combined environment analysis.

**Additional file 4:** **Table S4.** The number of QTL with favorable alleles contributed by five different parents.

## Data Availability

The SNP array data used in this study was submitted to OMIX (https://ngdc.cncb.ac.cn/omix/), and could be accessed using accession ID OMIX001002.

## Abbreviations

NAM: Nested association mapping
QTL: Quantitative trait loci
RIL: Recombinant inbred lines
HMW-GS: High-molecular-weight glutenin subunits
LMW-GS: Low-molecular-weight glutenin subunits
MAS: Maker-assisted selection
AACC: American Association of Cereal Chemists
BLUP: Best linear unbiased prediction
ANOVA: Analysis of variance
JICIM: Joint inclusive composite interval mapping
QG: QTL cluster
Add: Additive effects
MlPT: Midline peak time
MlPH: Midline peak height
PkWd: Peak width
WdEm: Width at eight minutes
YZ: Yanzhan 1
HU: Hussar
YN: Yunnanxiaomai
YT: Yutiandaomai
CY: Chayazheda 29
